# Vocal Cues to Male Physical Formidability

**DOI:** 10.3389/fpsyg.2022.879102

**Published:** 2022-07-05

**Authors:** Alvaro Mailhos, Damián Amaro Egea-Caparrós, Cristina Guerrero Rodríguez, Mario Luzardo, Nansi Dilyanova Kiskimska, Francisco Martínez Sánchez

**Affiliations:** ^1^Facultad de Psicología, Universidad de la República, Montevideo, Uruguay; ^2^Centro de Experimentación e Innovación Social, Universidad de la República, Montevideo, Uruguay; ^3^Facultad de Psicología, Universidad de Murcia, Murcia, Spain; ^4^Facultad de Psicología, Universidad de Cádiz, Cádiz, Spain; ^5^Instituto Universitario de Investigación para el Desarrollo Social Sostenible, Universidad de Cádiz, Jerez de la Frontera, Spain; ^6^Centro Universitario Regional del Este, Universidad de la República, Maldonado, Uruguay

**Keywords:** voice pitch, physical formidability, fundamental frequency, handgrip strength, sexual selection

## Abstract

Animal vocalizations convey important information about the emitter, including sex, age, biological quality, and emotional state. Early on, Darwin proposed that sex differences in auditory signals and vocalizations were driven by sexual selection mechanisms. In humans, studies on the association between male voice attributes and physical formidability have thus far reported mixed results. Hence, with a view to furthering our understanding of the role of human voice in advertising physical formidability, we sought to identify acoustic attributes of male voices associated with physical formidability proxies. Mean fundamental frequency (*F*_0_), formant dispersion (*D*_*f*_), formant position (*P*_*f*_), and vocal tract length (*VTL*) data from a sample of 101 male voices was analyzed for potential associations with height, weight, and maximal handgrip strength (*HGS*). *F*_0_ correlated negatively with *HGS*; *P*_*f*_ showed negative correlations with *HGS*, height and weight, whereas *VTL* positively correlated with *HGS*, height and weight. All zero-order correlations remained significant after controlling for false discovery rate (*FDR*) with the Benjamini–Hochberg method. After controlling for height and weight—and controlling for FDR—the correlation between *F*_0_ and *HGS* remained significant. In addition, to evaluate the ability of human male voices to advertise physical formidability to potential mates, 151 heterosexual female participants rated the voices of the 10 strongest and the 10 weakest males from the original sample for perceived physical strength, and given that physical strength is a desirable attribute in male partners, perceived attractiveness. Generalized linear mixed model analyses—which allow for generalization of inferences to other samples of both raters and targets—failed to support a significant association of perceived strength or attractiveness from voices alone and actual physical strength. These results add to the growing body of work on the role of human voices in conveying relevant biological information.

## Introduction

Different sensory channels — e g., chemical, acoustic, visual, and tactile — are used by animals to broadcast and assess opportunity and risk in agonistic and sexual interactions. Vocalizations conveying relevant information about the bearer, e.g., sex, age, biological quality, emotional states, and attitudes, can be broadcast over long distances, in the dark, or when lines of sight are interrupted.

Animal acoustic signals often exhibit some degree of sexual dimorphism. The occurrence of anatomical and behavioral sex differences had long puzzled naturalists, and it was not until Darwin published his seminal book “The Descent of Man, and Selection in Relation to Sex” (Darwin, 1872/1981) that a plausible mechanism for such differences had been proposed. It should be noted though, that Wallace seemed to favor the action of natural selection in promoting sex-specific differences (Wallace, 1889). More recently, additional mechanisms to account for the origin of sexually dimorphic characters have been proposed, e.g., sex-specific ecological niches and sexual food competition (Slatkin, 1984); the causes of sexual dimorphism seem multiple and might interact in complex manners (for a review of the causes of sex dimorphism in primates, see Plavcan, 2001). In many primates, including humans, vocalizations are markedly sexually dimorphic and both sexual selection mechanisms originally proposed by Darwin (1872/1981)—i.e., mate selection and intrasexual competition—are thought to be a major influence in voice attributes dimorphism (Pisanski et al., 2014, 2018; Puts et al., 2016).

Body size and strength are key determinants of animal physical formidability (Fessler et al., 2012) – i.e., fighting ability or resource-holding potential – and therefore play a major role in social interactions. In normal circumstances, larger individuals have better chances of winning agonistic contests with conspecific competitors over limited resources, however, because resources are seldom worth the risk of open combat, most animal species rely on social hierarchies and dominance cues to avoid physical injury (Fitch and Hauser, 2003). In this direction, body size also serves as a visual cue to physical prowess and dominance in different animal species. This is also true for male humans — i.e., taller men are perceived as more leader-like (Blaker et al., 2013) and stronger (Sell et al., 2009) than shorter men. Likewise, in naturalistic environments, the former are granted higher social dominance (Stulp et al., 2015), and in cooperative groups, stronger men are readily granted a higher social status and are perceived as stronger leaders by others (Lukaszewski et al., 2016).

Given the physiological and anatomical constraints acting upon sound production, some acoustic attributes of animal vocalizations serve as cues to body size and physical strength in different animal species. In terrestrial mammals, vocal signals are produced in a two-step process. First, air from the lungs is pushed through the larynx causing the vibration of the laryngeal vocal folds, then the laryngeal vibrations are propagated into the air within the supralaryngeal structures where they are modified by the resonances of these elements (for a review, see Taylor et al., 2016).

Because larger animals — having larger, heavier vocal folds — are predicted to produce vocalizations with a lower *F*_0_, a negative correlation between *F*_0_ and body size has been proposed (Titze, 2000). Along these lines, Bowling et al. (2017) found a negative correlation between “head + body size” and vocalization frequencies across a range of carnivore and primate species. Similarly, Martin et al. (2017) showed that body weight — another operationalization of body size — is an important determinant of minimum vocalization frequency in different terrestrial and aquatic mammal species. Analogous results were also reported by Riede and Brown (2013) based on their analysis of *F*_0_ vs. body weight in different mammal species, and more recently, Aung et al. (2021a) also reported negative associations of *F*_0_ and *P*_*f*_ with both height and weight.

However, because larynx development is not constrained by overall body size in humans and a few other mammalian species, and vocal folds can grow out of proportion in males in response to increased levels of testosterone during puberty, it has been suggested that vocalization frequencies do not reliably correlate with body size in those species (Fitch, 2000). Indeed, previous studies on the relationship between human *F*_0_ and body size have reported mixed results. A recent meta-analysis on the relationship between height and diverse acoustic attributes of human voice found that several *VTL* estimates based on formant frequencies accounted for up to 10% of the height and weight variance, while *F*_0_ accounted for less than 2% of those variances (Pisanski et al., 2016).

Thus far, relatively few studies have focused on the association between directly measured physical strength and voice attributes. While some studies reported a significant negative correlation of male physical strength with *F*_0_ (Hodges-Simeon et al., 2014), *P*_*f*_ (Puts et al., 2012; Hodges-Simeon et al., 2014), and the standard deviation of the fundamental frequency (*F_0_SD*; Puts et al., 2012), other studies, failed to find a significant relationship between physical strength and *F*_0_ (Sell et al., 2010; Atkinson et al., 2012; Smith et al., 2017; Han et al., 2018) or an association of physical strength with *F_0_SD*, *D*_*f*_, *P*_*f*_, or *VTL* (Han et al., 2018).

Similarly, research into the relationship between strength ratings from male voice stimuli and actual strength has also thus far provided mixed results. Puts et al. (2012) found a relationship between *F*_0_ and strength perceived from male voices, while Sell et al. (2010) observed a relationship between strength ratings from male voices and actual handgrip strength, suggesting the existence of vocal cues to physical strength, strength proxies, or covariates. In turn, Rosenfield et al. (2020) found that experimentally lowering the *F*_0_ of men voices increased the perception of fighting ability from voices alone in the Tsimané, a small-scale Amazonian society. Along these lines, in a recent meta-analysis of previous studies, Aung and Puts (2019) concluded that *F*_0_ showed a marginal negative correlation with upper-body strength ($\bar{r}$ = −0.07, *p* = 0.028).

The mixed results of those studies which explored the association between *F*_0_ and physical formidability (see above), and the low magnitude of this association whenever found, led some authors (Han et al., 2018; Armstrong et al., 2019; and notably Feinberg et al., 2019) to propose that *F*_0_ does not signal formidability. Because a trend to associate *F*_0_ and formidability has been reported in many studies, these authors suggest that this tendency reflects a general bias to associate lower frequency sounds with larger sound sources. Puts and Aung (2019), on the other side of the debate, claim that regardless of the way an association between low voice pitch and formidability might have originated, such an association would only be maintained if it were an honest signal. Furthermore, it has recently been shown that F_0_ mediates the relationship between objective and perceived dominance, that is that listeners perceive formidable men as being more formidable in part because of their voice pitch (Aung et al., 2021b).

In relation to the apparent association of listeners’ perceptions of physical formidability and actual biological attributes, in absence of evident associations of acoustic attributes and formidability proxies, Kleisner et al. (2021), have also suggested that physical strength—and most likely other biological attributes—cannot be predicted solely by one acoustic variable, but rather by a combination of multiple interacting acoustic parameters.

Interestingly, in recent years researchers have begun to explore the association of non-verbal vocalizations in humans (e.g., roars, screams, grunts, and laughs) and their role in signaling different biological qualities. Listeners seem to be able to accurately judge inter-individual differences in strength from roars, while no single acoustic attribute seem to be associated with actual strength (Raine et al., 2019; Šebesta et al., 2019; Kleisner et al., 2021). In addition, roars seem to better predict strength than aggressive or normal speech (Raine et al., 2019; Šebesta et al., 2019; Kleisner et al., 2021). Non-linear phenomena appear to play an important part in the communication role of non-verbal vocalizations (Anikin et al., 2021; Kleisner et al., 2021) by means of lowering perceived voice pitch, and causing vocalizers to sound larger, more formidable and more aggressive (Anikin et al., 2021).

This study is aimed at expanding on previous findings on the association of different sexually dimorphic attributes of male voices — *F*_0_, *D*_*f*_, *P*_*f*_, and *VTL* — with height, weight and physical strength, three key determinants of physical formidability, and thus, contributing to the current debate. Here, we also explore whether perceptual adaptations may have enabled the assessment of physical formidability by females based on male voices alone. The latter study was designed to test the hypothesis of a positive association between male speakers’ strength and strength perception from their voices by female raters.

## Materials and Methods

### Anthropometry and Acoustic Attributes

#### Anthropometric Measurements and Voice Recordings

One hundred one self-reported heterosexual male students from a mid-sized Spanish university [median age = 20.48 years, interquartile range (*IQR*) = 2.36 years] participated in this study in exchange for course credit. The procedure was approved by the Local Research Ethics Board, and all participants gave written informed consent. Height was measured using a Seca 217 stadiometer and weight was measured on an electronic scale (Rowenta Bodymaster) using standard procedures. Handgrip strength was measured in both hands using a hydraulic dynamometer (Saehan SH5001), taking three measurements for each hand, alternating sides. Because 50% of left-handed people and 9% of right-handed people are weaker in their dominant hand than in their non-dominant hand (Crosby and Wehbé, 1994), here, maximal handgrip strength, i.e., regardless of hand and handedness, was used for the analysis.

The male participants provided voice recordings by reading the following text: “Hace unos días te llamaron para tener una entrevista de trabajo en una importante empresa internacional” (A few days ago you were called in for a job interview at a major international company). All participants were native speakers of Spanish. Recordings were made with an AKG D3700S cardioid microphone and a Fostex FR-2LE recorder at a sampling rate of 44,100 Hz and 16-bit quantization. All recordings were made by the same researcher keeping all conditions unchanged in a quiet, non-soundproofed room, locating the microphone 8 cm away from the speaker’s mouth at an angle of 45° to avoid aerodynamic noise. Recordings were saved as uncompressed .wav files.

#### Spectrographic Analysis of Male Voices

Each recording was analyzed using Praat version 5.2.27 software (Boersma and Weenink, 2017). The fundamental frequency was calculated using acoustic periodicity detection based on autocorrelations, i.e., correlating of a time-domain signal with itself; this technique is more accurate, noise-independent, and robust than alternative methods like those based on cepstrum or combs (Boersma and Weenink, 2017). A floor of 75 Hz and a ceiling of 300 Hz, with a Hanning window length of 0.01 s, were used following programmer’s recommendations (Boersma and Weenink, 2017). *F*_0_ was calculated according to programmer’s instructions; F1 through F4 were measured using Praat as described by Valentova et al. (2019), while *D*_*f*_, *P*_*f*_, and *VTL* were calculated as described by Fitch (1997), Reby and McComb (2003), and Puts et al. (2012), respectively.

### Voice Ratings

#### Vocal Stimuli for Physical Strength, and Attractive Ratings

Voice recordings from the 10 weakest and 10 strongest males from the same sample were used as stimuli in the voice assessment study. Groups do differ significantly in height, weight, *HGS* and *VTL*, while they do not present significant differences in age, *F*_0_, *D*_*f*_, and *P*_*f*_ (see Table 1).

**TABLE 1:** Mann–Whitney *U*-test results.

	Weakest males mean rank	Strongest males mean rank	U	Z	*P*
Age	11.8	9.20	37.00	−0.982	0.326
Height	7.35	13.65	18.50	−2.381	0.017
Weight	7.80	13.20	23.00	−2.041	0.041
HGS	5.50	15.50	0.00	−3.779	<0.001
F_0_	12.10	8.90	34.00	−1.209	0.226
D_*f*_	12.50	8.50	30.00	−1.512	0.130
P_*f*_	12.60	8.40	29.00	−1.587	0.112
VTL	7.70	13.3	22.00	−2.117	0.034

#### Female Raters

One hundred fifty-one self-reported heterosexual female students from a mid-sized Spanish university (median age = 20.16 years, IQR = 2.11) who reported no chronic or acute hearing impairment participated in the study in exchange for course credit. Voice recordings were presented in random order by means of headphones, and participants were asked to rate each voice for perceived physical strength and perceived attractiveness on a 4-point ordinal scale (1 = physically weak, 4 = physically strong; 1 = unattractive, 4 = attractive). All raters gave written informed consent and assessed all voice stimuli.

#### Statistical Analyses

All data analyses were conducted with R software (v4.1.0; R Core Team, 2021). Shapiro–Wilk tests of normality indicated that males’ age, anthropometric variables (except body weight) and voice variables were non-normally distributed. Associations between anthropometric variables and acoustic attributes were explored by means of Spearman rank correlation analysis. All tests were two-tailed, and the significance level was set to α = 0.05. In order to avoid false positive findings, the Benjamini and Hochberg (1995) false discovery rate (FDR) was used to correct multiple comparisons with FDR < 0.05 considered as statistically significant.

Given that previous studies in this field have shown inconsistent results, it was expected that if any association was to be observed, they would range from low to moderate in magnitude. In this study, the association of voice attributes with different proxies of physical formidability in a sample of 101 men was analyzed. While this study might be slightly underpowered, our sample size would allow to detect a correlation of 0.275 with a 0.80 statistical power. Bootstrapping provides an alternative to relying on underpowered samples. Thus, the significance of the correlations was also assessed through this method with 5,000 simulation iterations.

In the voice ratings study, to avoid random effects in raters and targets, linear mixed models were used to assess the relationship of measured handgrip strength with perceived physical strength and attractiveness from voices alone.

Data were analyzed using R statistical package version v4.1.0 (R Core Team, 2021) using the ordinal package (Christensen, 2019). Model parameters were estimated according to maximum likelihood using Laplace’s approximation (clmm function).

## Results

### Anthropometric Measurements and Vocal Frequencies

Descriptive statistics of the speakers’ anthropometric and acoustic variables used in this study are shown in Table 2 (see also Supplementary Tables 1, 2).

**TABLE 2:** Descriptive statistics for the male sample.

	Min	Max	Mean	SD	Median	MAD	IQR
Age (yrs)	17.98	26.11	20.77	1.77	20.52	1.85	2.36
Height (cm)	161.00	192.20	176.55	6.97	176.45	6.75	8.93
Weight (Kg)	53.50	114.40	73.36	11.68	72.35	10.08	13.28
HGS (Kg)	24.50	63.00	44.49	8.61	44.40	8.01	10.90
F_0_ (HZ)	94.28	167.23	120.51	14.88	121.00	18.25	22.50
D_*f*_ (Hz)	855.30	1284.39	1054.27	75.47	1057.92	67.32	92.62
P_*f*_	−1.29	1.28	0.00	0.63	−0.06	0.71	1.01
VTL (cm)	15.63	19.22	17.32	0.85	17.32	0.92	1.24

*SD, Standard deviation; MAD, median absolute deviation; IQR, interquartile range.*

In order to analyze the relationship between anthropometric measurements and the different acoustic attributes, Spearman rank correlation coefficients were calculated. Height showed a significant negative correlation with *P*_*f*_ and a positive correlation with *VTL* but did not significantly correlate with *F*_0_ or *D*_*f*_. Similarly, weight — another operationalization of body size — showed significant negative correlations with *D*_*f*_ and *P*_*f*_, and a positive correlation with *VTL*, but did not show a significant correlation with *F*_0_. *HGS* showed significant negative correlations with *F*_0_, *P*_*f*_ and a positive correlation with the estimated *VTL*, but did not correlate with *D*_*f*_.

None of the acoustic attributes of male voices, nor the anthropometric speaker variables showed any significant correlations with age. As expected, height, weight and *HGS* were significantly correlated with each other, as were some of the acoustic attributes of male voices among themselves. The complete correlation matrix is shown in Table 3.

**TABLE 3:** Zero order Spearman correlations of anthropometric correlates of physical formidability and acoustic variables.

	Age	Height	Weight	HGS	F_0_	D_f_	P_f_	VTL
Age	–	−0.03 (*p* = 0.787)	0.07 (*p* = 0.502)	−0.07 (*p* = 0.5)	0.04 (*p* = 0.729)	−0.09 (*p* = 0.355)	−0.06 (*p* = 0.534)	0.11 (*p* = 0.787)
Height		–	0.57 (*p* < 0.001)	0.34 (*p* < 0.001)	−0.1 (*p* = 0.329)	−0.18 (*p* = 0.078)	−0.27 (*p* = 0.006)	0.27 (*p* = 0.006)
Weight			–	0.33 (*p* < 0.001)	−0.03 (*p* = 0.769)	−0.25 (*p* = 0.013)	−0.36 (*p* < 0.001)	0.31 (*p* = 0.002)
HGS				–	−0.26 (*p* = 0.009)	−0.23 (*p* = 0.018)	−0.28 (*p* = 0.004)	0.28 (*p* = 0.004)
F_0_					–	0.02 (*p* = 0.835)	0.05 (*p* = 0.612)	−0.02 (*p* = 0.865)
D_f_						–	0.56 (*p* < 0.001)	−0.88 (*p* < 0.001)
P_f_							–	−0.84 (*p* < 0.001)
VTL								–

After correcting for false discovery rate using the Benjamini–Hochberg method, all significant zero-order correlations remained significant (see Supplementary Table 3). In addition, because our study was slightly underpowered (see section “Materials and Methods”), the confidence intervals of all correlations were calculated through bootstrapping. All significant correlations were supported by this analysis (see Supplementary Table 4).

To control for any effect of body size (i.e., height and weight) on the correlation of *HGS* with the acoustic variables, and vice versa, partial Spearman rank correlations were calculated. After controlling for height and weight, the correlation of *HGS* with *F*_0_ and *VTL* remained significant, with very little variation in the correlation coefficients and *p*-values (see Table 4), while the correlation between *HGS* and *P*_*f*_ bordered significance [*r*_(101)_ = −0.29, *p* = 0.052] when controlling for the aforementioned variables. In turn, when the effects of *HGS* and weight are controlled for, the correlations of height with *P*_*f*_ and *VTL* become non-significant; and when the effects of *HGS* and height are partialled out, only the correlation between weight and *P*_*f*_ remained significant (see Table 4). However, only the partial correlation between *F*_0_ and *HGS*—controlling for height and weight—remained significant after adjusting for the false discovery rate using the Benjamini–Hochberg method (see Supplementary Table 5).

**TABLE 4:** Partial Spearman correlations of anthropometric correlates of physical formidability with acoustic attributes of male voices.

	F_0_	D_f_	P_f_	VTL
HGS	−0.25	−0.19	−0.20	0.21
(controlling for height and weight)	(*p* = 0.012)	(*p* = 0.068)	(*p* = 0.052)	(*p* = 0.038)
Height	−0.05	−0.01	−0.05	0.08
(controlling for strength and weight)	(*p* = 0.633)	(*p* = 0.939)	(*p* = 0.597)	(*p* = 0.410)
Weight	0.08	−0.15	−0.22	0.16
(controlling for strength and height)	(*p* = 0.442)	(*p* = 0.140)	(*p* = 0.029)	(*p* = 0.127)

### Physical Strength and Attractiveness Assessments From Male Voices

Table 5 summarizes the descriptive statistics of physical strength and attractiveness ratings for male voices provided by female raters.

**TABLE 5:** Female ratings of male voices.

	Weakest males		Strongest males	
	Mean	SD	Mean	SD
Strength ratings	2.28	0.39	2.59	0.35
Attractiveness ratings	2.12	0.40	2.42	0.42

*SD, standard deviation.*

Linear mixed model analyses were used to identify a possible relationship between speakers’ handgrip strength and physical strength perceived from male voices. These models contribute to avoiding false-positives by accounting for random variance between raters and targets, and allow the generalization of inferences to other raters and targets (e.g., Durkee, 2019). Given that strength ratings can take values between 1 and 4 (4 ordinal levels), a mixed multilevel logistic regression with cumulative logit link function was used to model handgrip strength.

Three models were considered, as follows: Model 1—the maximal model for our design—which allowed random intercepts for both raters and targets and random slopes for raters, while Model 2 allowed random intercepts for both raters and targets and fixed slopes, and Model 3 allowed random intercepts and slopes for raters only, but not for targets, that is, by considering targets as fixed effects, thus Model 3 only allowed to evaluate the ability of raters to accurately appraise strength based on this particular set of voice stimuli. Random effects were significant for all three models (see Table 6).

**TABLE 6:** Standard deviation of raters’ and targets’ random effects.

		Group-level effects	SD
Strength ratings	Model 1	Rater (Intercept)	1.01
		Rater (Group slope)	0.49
		Target (Intercept)	1.35
	Model 2	Rater (Intercept)	0.88
		Target (Intercept)	1.33
	Model 3	Rater (Intercept)	0.70
		Rater (Group slope)	0.11
Attractiveness ratings	Model 1	Rater (Intercept)	0.91
		Rater (Group slope)	0.15
		Target (Intercept)	1.28
	Model 2	Rater (Intercept)	0.95
		Target (Intercept)	1.28
	Model 3	Rater (Intercept)	0.70

*SD, Random effect standard deviation using ordinal cumulative mixed models.*

Models 1, 2, and 3 were assessed using the corrected Akaike’s Information Criterion (*AICc*), *ΔAICc*—the difference between the test model *AICc* and the *AICc* of best fitting model— and *AICc* weights [*w_*i*_(AIC)*]—the conditional probabilities for each model being the best model (Wagenmakers and Farrell, 2004). Based on these criteria, Model 1 appears to be the most plausible model (see Table 7).

**TABLE 7:** Information criteria for the general linear mixed models.

	AICc	ΔAICc	Df	wi(AICc)
**Strength ratings**				
Model 1	6256.43	0.00	8.00	0.93
Model 2	6261.62	5.19	6.00	0.07
Model 3	7308.61	1052.18	5.00	0.00
**Attractiveness ratings**				
Model 2	6607.76	0.00	6.00	0.86
Model 1	6611.41	3.66	8.00	0.14
Model 3	7602.35	994.59	5.00	0.00

For Model 1, the group (weakest vs. strongest men) coefficient did not reach statistical significance (*b* = 0.93, *p* = 0.126), therefore perceived strength from voices only is not significantly associated with actual strength.

Following the same approach, using attractiveness ratings based on male voices alone the analysis showed that random effects were significant for all three models (see Table 6). Given that the *ΔAICc* between Models 2 and 1 is 3.66, both models could be considered, however, *wi(AICc)* for model 2 is far larger than *wi(AICc)* (0.86 vs. 0.14), thus, Model 2 is the most plausible model (see Table 7).

The group (weakest vs. strongest men) coefficient for Model 2 did not reach significance (*b* = 0.78, *p* = 0.176). Thus, target strength group — i.e., weakest vs. strongest males — does not have a significant effect on the attractiveness ratings by female raters.

## Discussion

Here, we found that *F*_0_, one of the most noticeable attributes of human voice, is negatively correlated with handgrip strength in males, and that this correlation remains significant after controlling for false discovery rate by Benjamini–Hochberg and potential confounders (height and weight). This finding is in line with the observations of Hodges-Simeon et al. (2014), who reported a negative association between *F*_0_ and upper-body strength in a sample of peripubertal Tsimane males, but seems to contradict other studies which failed to find an association of voice pitch and physical strength (Sell et al., 2010; Atkinson et al., 2012; Smith et al., 2017; Han et al., 2018). Our results are also consistent with the conclusions of a recent meta-analysis. While the zero-order correlation between *F*_0_ and physical strength in our study was moderate in size (*r* = −0.26, *p* = 0.009), this association appeared to be stronger than the correlation reported in the meta-analysis conducted by Aung and Puts (2019) in which the authors found a marginal association between male *F*_0_ and upper-body strength ($\bar{r}$ = −0.07, *p* = 0.028). It should be noted that, whereas the studies included in Aung and Puts’ meta-analysis used a composite measurement of upper-body strength, in our study direct measurements of handgrip strength alone were used. Such a methodological difference may have contributed to apparent differences over the correlation between *F*_0_ and physical strength. In addition, the studies included in Aung and Puts’ meta-analysis may not have been statistically robust enough to assert a moderate correlation; 9 of the 11 male samples included in that meta-analysis ranged from 8 to 63 participants only, and/or exhibited substantial age heterogeneity. For instance, an Andean sample consisted of men aged between 15 and 71 (*N* = 20, *M* = 34.8, *SD* = 19.10), while, in another study, the age of Tsimane participants ranged between 19 and 68 years (*N* = 49, *M* = 35.8, *SD* = 13.5) — compared to a low age dispersion in the sample used in the current study (median age = 20.48 years, *IQR* = 2.36).

Thus, with regard to the debate as to whether *F*_0_ actually signals formidability or simply reflects a general tendency to associate lower frequency sounds with larger bodies, our results seem to favor the former position, in that *F*_0_ correlates negatively with physical strength, one of the key components of physical formidability.

While we found a significant positive a zero-order correlation between *VTL* and *HGS* that remains significant after we control for height and weight (*r* = 0.21, *p* = 0.038), our results fail to show an association between *VTL* and *height* when we control for the contribution of *strength* and *weight* (*r* = 0.08, *p* = 0.410). It should be noted that previous associations between *VTL* and *height* were based on zero-order correlations (Pisanski et al., 2016), while in this study we controlled for potentially confounding variables. However, only the correlation between *F*_0_ and *HGS* remains significant after controlling for false discovery rate and potential confounders.

In order to assess the non-verbal communicational value of male voices, the voices of the 10 weakest and 10 strongest males were rated by the female subjects for perceived strength and attractiveness. Here, actual physical strength failed to show a significant effect on perceived strength or attractiveness ratings from male voices given by heterosexual female subjects. This negative result seems to contradict previous studies, where the authors found that listeners could assess physical strength from voices (Sell et al., 2010; Raine et al., 2018, 2019) or roars (Raine et al., 2018, 2019). However, our results are in line with other studies which found no association between physical strength—a key determinant of fighting ability—and perceived fighting ability (Šebesta et al., 2019), between actual threat potential—derived from the measures of their upper-body strength, height, and weight—and perceived threat potential (Han et al., 2017), or an association between self-reported fighting ability and perceived fighting ability (Doll et al., 2014)—which could be assumed to be related, at least in part, to physical strength.

The different outcomes of the studies above might reflect differences in fitness variables used in the various studies (handgrip strength, composite measures of fighting ability or threat potential), and/or procedure differences (ratings of perceived fighting ability, threat potential or strength). Thus, it is clear that the true nature of the association of actual and perceived strength—or its proxies—is still unclear; and a standardized approach to this issue will help comparison among different studies.

Unlike previous studies, here the effect of actual speaker strength on perceived strength ratings elicited by male voice stimuli was assessed by ordinal linear mixed models so as to avoid inflated false-positives due to random variance between raters and stimuli. The treatment of raters and stimuli as random effects increases the accurateness of extrapolating statistical inferences to other samples of raters and stimuli (Judd et al., 2012; Durkee, 2019). While this approach allows for the generalization of the results to other samples, it is also more strict than fixed effects models; therefore, this might contribute to explain the differences observed in different studies.

Intersexual selection and intrasexual competition—the two original mechanisms of sexual selection proposed by Darwin (1872/1981)—may both act upon the same sexually dimorphic characters (Berglund et al., 1996), that is, secondary sex characters may play a role both in intrasexual competition, and also serve as cues in mate selection. While in this study we analyzed the potential of male voices in advertising physical strength to female raters—a desirable attribute and a potential indicator of biological quality—it will of interest to analyze whether there are sex-specific differences in assessing physical strength from auditory cues, e.g., if males are better in assessing physical strength from voices only; if so, this might be indicative of a greater role of male voices in intrasexual competition mechanisms.

Our research has some potential limitations and shortcomings. A larger sample would have been needed to provide sufficient statistical power to detect a low-to-moderate correlation between vocal frequency and body size (Pisanski et al., 2014). In the same vein, physical strength and attractiveness ratings from male voices provided by male raters – together with female ratings – would have helped to further understand the role of intrasexual competition and intersexual selection in the evolution of voice sexual dimorphism. Further, only a few acoustic attributes of human voice were studied here. In this regard, the analysis of prosodic attributes of human voice may prove essential to effectively understanding the role of human voice in non-verbal communication.

## Data Availability Statement

The raw data supporting the conclusions of this article will be made available by the authors, without undue reservation.

## Ethics Statement

The studies involving human participants were reviewed and approved by the Comité de Ética de Investigación, Facultad de Psicología, Universidad de Murcia, Spain. The patients/participants provided their written informed consent to participate in this study.

## Author Contributions

AM and FM proposed and designed the study. AM, FM, DE-C, CG, and NK collected the data. ML conducted the data analysis. AM, FM, and ML wrote the manuscript. All authors contributed to the article and approved the submitted version.

## Conflict of Interest

The authors declare that the research was conducted in the absence of any commercial or financial relationships that could be construed as a potential conflict of interest.

## Publisher’s Note

All claims expressed in this article are solely those of the authors and do not necessarily represent those of their affiliated organizations, or those of the publisher, the editors and the reviewers. Any product that may be evaluated in this article, or claim that may be made by its manufacturer, is not guaranteed or endorsed by the publisher.
