# Supplementary material for: Vocal Cues to Male Physical Formidability
Source: Front Psychol. 2022 Jul 5;13:879102. doi: 10.3389/fpsyg.2022.879102 (PMC9294471; doi:10.3389/fpsyg.2022.879102)
Supplement: Supplementary file 1 [file Table_1.docx]

Supplementary Materials Table 1

*Main descriptive statistics for the 10 weakest men*

|  | Min | Max | Mean | SD | Median | MAD | IQR |
| --- | --- | --- | --- | --- | --- | --- | --- |
| Age (yrs) | 19.04 | 26.11 | 21.48 | 2.31 | 20.71 | 1.73 | 3.53 |
| Height (cm) | 161.00 | 182.40 | 172.06 | 7.66 | 174.65 | 8.30 | 12.18 |
| Weight (Kg) | 54.10 | 84.40 | 67.60 | 10.05 | 66.75 | 12.45 | 15.03 |
| HGS (Kg) | 24.50 | 32.00 | 29.15 | 3.01 | 30.05 | 2.89 | 4.98 |
| F_0_ (Hz) | 100.69 | 152.79 | 126.34 | 16.24 | 123.64 | 13.29 | 20.96 |
| D_f_ (Hz) | 959.45 | 1153.59 | 1062.41 | 70.31 | 1094.27 | 64.76 | 116.34 |
| P_f_ | -0.36 | 0.65 | 0.22 | 0.36 | 0.21 | 0.46 | 0.61 |
| VTL (cm) | 16.47 | 18.06 | 17.07 | 0.63 | 16.68 | 0.21 | 1.06 |

*Note*. SD: Standard deviation; MAD: median absolute deviation; IQR: interquartile range.
