# Supplementary material for: Vocal Cues to Male Physical Formidability
Source: Front Psychol. 2022 Jul 5;13:879102. doi: 10.3389/fpsyg.2022.879102 (PMC9294471; doi:10.3389/fpsyg.2022.879102)
Supplement: Supplementary file 2 [file Table_2.docx]

Supplementary Materials Table 2

*Main descriptive statistics for the 10 strongest men*

|  | Min | Max | Mean | SD | Median | MAD | IQR |
| --- | --- | --- | --- | --- | --- | --- | --- |
| Age (yrs) | 18.64 | 21.92 | 20.29 | 1.37 | 20.27 | 2.27 | 3.01 |
| Height (cm) | 165.00 | 188.10 | 179.21 | 7.46 | 181.60 | 2.22 | 2.63 |
| Weight (Kg) | 59.90 | 102.70 | 80.37 | 14.43 | 80.65 | 18.46 | 22.93 |
| HGS (Kg) | 55.50 | 63.00 | 60.15 | 2.29 | 60.50 | 1.11 | 1.38 |
| F0 (Hz) | 94.28 | 156.54 | 116.63 | 17.93 | 113.64 | 15.69 | 19.30 |
| D_f_ (Hz) | 935.58 | 1075.23 | 1022.54 | 51.26 | 1034.15 | 56.86 | 80.52 |
| Pf | -0.80 | 1.22 | -0.10 | 0.67 | -0.25 | 0.72 | 0.98 |
| VTL (cm) | 16.78 | 18.84 | 17.66 | 0.68 | 17.58 | 0.90 | 1.09 |

*Note*. SD: Standard deviation; MAD: median absolute deviation; IQR: interquartile range.
