# Supplementary material for: Vocal Cues to Male Physical Formidability
Source: Front Psychol. 2022 Jul 5;13:879102. doi: 10.3389/fpsyg.2022.879102 (PMC9294471; doi:10.3389/fpsyg.2022.879102)
Supplement: Supplementary file 3 [file Table_3.docx]

Supplementary Materials Table 3

*Benjamini-Hochberg´s false discovery rate analysis for zero-order correlations*

|  | p-value | Index | d*i/n | p < d*i/n |
| --- | --- | --- | --- | --- |
| D_f_ & VTL | 0.000 | 1 | 0.002 | TRUE |
| P_f_ & VTL | 0.000 | 2 | 0.004 | TRUE |
| Height & Weight | 0.000 | 3 | 0.005 | TRUE |
| D_f_ & P_f_ | 0.000 | 4 | 0.007 | TRUE |
| Weight & P_f_ | 0.000 | 5 | 0.009 | TRUE |
| Height & HGS | 0.000 | 6 | 0.011 | TRUE |
| Weight & HGS | 0.001 | 7 | 0.013 | TRUE |
| Weight & VTL | 0.002 | 8 | 0.014 | TRUE |
| HGS & P_f_ | 0.004 | 9 | 0.016 | TRUE |
| HGS & VTL | 0.004 | 10 | 0.018 | TRUE |
| Height & P_f_ | 0.006 | 11 | 0.020 | TRUE |
| Height & VTL | 0.006 | 12 | 0.021 | TRUE |
| HGS & F_0_ | 0.010 | 13 | 0.023 | TRUE |
| Weight & D_f_ | 0.013 | 14 | 0.025 | TRUE |
| HGS & D_f_ | 0.018 | 15 | 0.027 | TRUE |
| Height & Df | 0.078 | 16 | 0.029 | FALSE |
| Age & VTL | 0.300 | 17 | 0.030 | FALSE |
| Height & F_0_ | 0.329 | 18 | 0.032 | FALSE |
| Age & D_f_ | 0.355 | 19 | 0.034 | FALSE |
| Age & HGS | 0.500 | 20 | 0.036 | FALSE |
| Age & Weight | 0.502 | 21 | 0.038 | FALSE |
| Age & P_f_ | 0.534 | 22 | 0.039 | FALSE |
| F0 & P_f_ | 0.612 | 23 | 0.041 | FALSE |
| Age & F_0_ | 0.729 | 24 | 0.043 | FALSE |
| Weight & F_0_ | 0.769 | 25 | 0.045 | FALSE |
| Age & Height | 0.787 | 26 | 0.046 | FALSE |
| F_0_ & D_f_ | 0.835 | 27 | 0.048 | FALSE |
| F_0_ & VTL | 0.865 | 28 | 0.050 | FALSE |

*Note*. *d*: 0.05; *i*: index; *n*: total number of comparisons.
