# Supplementary material for: Vocal Cues to Male Physical Formidability
Source: Front Psychol. 2022 Jul 5;13:879102. doi: 10.3389/fpsyg.2022.879102 (PMC9294471; doi:10.3389/fpsyg.2022.879102)
Supplement: Supplementary file 4 [file Table_4.docx]

Supplementary Materials Table 4

*Boostrap confidence intervals of zero-order correlation coefficients.*

| Correlation | 95% Confidence Interval | | |
| --- | --- | --- | --- |
|  | Lower limit |  | Upper limit |
| Age & Height | -0.24 |  | 0.175 |
| Age & Weight | -0.138 |  | 0.277 |
| Age & HGS | -0.267 |  | 0.138 |
| Age & F_0_ | -0.179 |  | 0.251 |
| Age & D_f_ | -0.311 |  | 0.124 |
| Age & P_f_ | -0.263 |  | 0.143 |
| Age & VTL | -0.11 |  | 0.315 |
| Height & Weight * | 0.42 |  | 0.691 |
| Height & HGS * | 0.16 |  | 0.508 |
| Height & F_0_ | -0.301 |  | 0.114 |
| Height & D_f_ | -0.36 |  | 0.012 |
| Height & P_f_ * | -0.459 |  | -0.076 |
| Height & VTL * | 0.086 |  | 0.44 |
| Weight & HGS * | 0.139 |  | 0.509 |
| Weight & F_0_ | -0.22 |  | 0.165 |
| Weight & D_f_ * | -0.425 |  | -0.052 |
| Weight & Pf * | -0.516 |  | -0.174 |
| Weight & VTL * | 0.128 |  | 0.459 |
| HGS & F_0_ * | -0.435 |  | -0.061 |
| HGS & D_f_ * | -0.428 |  | -0.037 |
| HGS & P_f_ * | -0.452 |  | -0.102 |
| HGS & VTL * | 0.096 |  | 0.455 |
| F_0_ & D_f_ | -0.177 |  | 0.21 |
| F_0_ & P_f_ | -0.164 |  | 0.261 |
| F_0_ & VTL | -0.213 |  | 0.182 |
| D_f_ & P_f_ * | 0.405 |  | 0.69 |
| D_f_ & VTL * | -0.929 |  | -0.807 |
| P_f_ & VTL * | -0.884 |  | -0.768 |
| *Note*. * Indicates significant correlations. | | | |
