# Supplementary material for: Vocal Cues to Male Physical Formidability
Source: Front Psychol. 2022 Jul 5;13:879102. doi: 10.3389/fpsyg.2022.879102 (PMC9294471; doi:10.3389/fpsyg.2022.879102)
Supplement: Supplementary file 5 [file Table_5.docx]

Supplementary Materials Table 5

*Benjamini-Hochberg´s false discovery rate analysis for partial correlations*

|  |  | p-value | index | d*i/n | p < d*i/n |
| --- | --- | --- | --- | --- | --- |
| Controlling for height and weight | HGS & F_0_ | 0.012 | 1 | 0.01 | TRUE |
|  | HGS & VTL | 0.038 | 2 | 0.03 | FALSE |
|  | HGS & P_f_ | 0.052 | 3 | 0.04 | FALSE |
|  | HGS & D_f_ | 0.068 | 4 | 0.05 | FALSE |
|  |  |  |  |  |  |
| Controlling for strength and weight | Height & VTL | 0.410 | 1 | 0.01 | FALSE |
|  | Height & P_f_ | 0.597 | 2 | 0.03 | FALSE |
|  | Height & F_0_ | 0.633 | 3 | 0.04 | FALSE |
|  | Height & D_f_ | 0.939 | 4 | 0.05 | FALSE |
|  |  |  |  |  |  |
| Controlling for height and strength | Weight & P_f_ | 0.029 | 1 | 0.01 | FALSE |
|  | Weight & VTL | 0.127 | 2 | 0.03 | FALSE |
|  | Weight & D_f_ | 0.140 | 3 | 0.04 | FALSE |
|  | Weight & F_0_ | 0.442 | 4 | 0.05 | FALSE |

*Note*. *d*: 0.05; *i*: index; *n*: total number of comparisons.
